# Leveraging connections between family planning and nutrition to improve the health of women

**DOI:** 10.1136/bmjgh-2024-017481

**Published:** 2026-04-13

**Authors:** Uttara Partap, Sachin Shinde, Ilana Rachel Cliffer, Dongqing Wang, Cara Yelverton, Moussa Ouédraogo, Innocent Yusufu, Ourohire Millogo, Mary Mwanyika-Sando, Ali Sie, Iqbal Shah, Wafaie Fawzi

**Affiliations:** 1 Department of Global Health and Population, Harvard T H Chan School of Public Health, Boston, Massachusetts, USA; 2 Department of Global and Community Health, George Mason University College of Public Health, Fairfax, Virginia, USA; 3 Nouna Health Research Center, Nouna, Boucle du Mouhoun Region, Burkina Faso; 4 Africa Academy for Public Health, Dar es Salaam, Tanzania; 5 Research Institute of Health Sciences, Ouagadougou, Centre Region, Burkina Faso

**Keywords:** Global Health, Nutrition, Public Health, Maternal health

## Abstract

The global burden of malnutrition and unmet need for family planning (FP) among women of reproductive age remains high. Leveraging the epidemiological and programmatic links between FP and nutrition could help improve outcomes in both domains for women, with wider benefits to health and well-being. Here, we draw on findings from our recently concluded Family Planning and Nutrition Project, which synthesised epidemiological and programmatic linkages between FP and nutrition, alongside other related literature, to argue that limited research connecting FP and nutrition is constraining concrete investments in this area. We identify three key areas for evidence generation with the potential to advance women’s health: examining the impact of hormonal contraceptives to address anaemia caused by heavy menstrual bleeding; strengthening postpartum FP-nutrition service integration and capitalising on social protection programmes to deliver FP and nutrition-related services. Robust evidence from large-scale implementation studies focusing on these areas will be fundamental to reliably establishing the value of such approaches —including effectiveness, cost-effectiveness and key indicators such as acceptability and feasibility —and drawing further resource commitments. Investment in these approaches will help address the unique needs of women across the life course and contribute to improving women’s health outcomes globally.

**SUMMARY BOX**

- Women have overlapping family planning (FP) and nutrition-related needs across their life course, and meeting these needs together has the potential to enhance benefits to women’s health.
- FP use may improve nutritional status through direct physiological effects as well as through controlling the timing and number of pregnancies and increasing the ability to take advantage of educational and socioeconomic opportunities and resources; on the other hand, nutritional status is a key determinant of reproductive health, fertility and fetal survival , and thus an indirect determinant of patterns of FP use.
- There is scope to leverage these FP-nutrition connections to ensure the greatest possible benefit for women’s health through investing in research examining FP as a tool to improve nutritional status by targeting heavy menstrual bleeding, strengthening FP and nutrition services in the postpartum period and building on social protection programmes to co-deliver FP and nutrition services.
- Maximising benefits to women’s health through capitalising on FP-nutrition connections represents an important contribution to improving women’s well-being and empowerment globally.

## Introduction

Ensuring access to optimal family planning (FP) and nutrition among women is key to advancing gender equality, women’s health and overall population health and development.^1^ Elements of both are embedded in the Sustainable Development Goals, including targets 2.2 (end all forms of malnutrition), 3.7 and 5.6 (ensure universal access to sexual and reproductive healthcare services and rights).^2^ However, progress towards ensuring appropriate access to FP and nutrition among women of reproductive age (WRA) remains slow, with the greatest gaps remaining in low- and middle-income countries (LMICs). In 2019, over 160 million WRA were estimated to have an unmet need for contraceptives, with over half of these residing in South Asia and sub-Saharan Africa (SSA).^3^ Recent estimates indicate that 30% – 35 % of WRA globally have anaemia,^4^ while about two-thirds of non-pregnant WRA globally (80 % in SSA) are estimated to have a deficiency of iron, zinc or folate.^5^ In 2022, underweight (Body Mass Index (BMI) < 18.5 kg/m^2^ ) prevalence among women was 7.6 %,^6^ and overweight or obesity prevalence was 43.9 %,^7^ with particularly substantial increases in overweight and obesity prevalence being observed and forecast in LMICs.^8^

**Figure 1:**
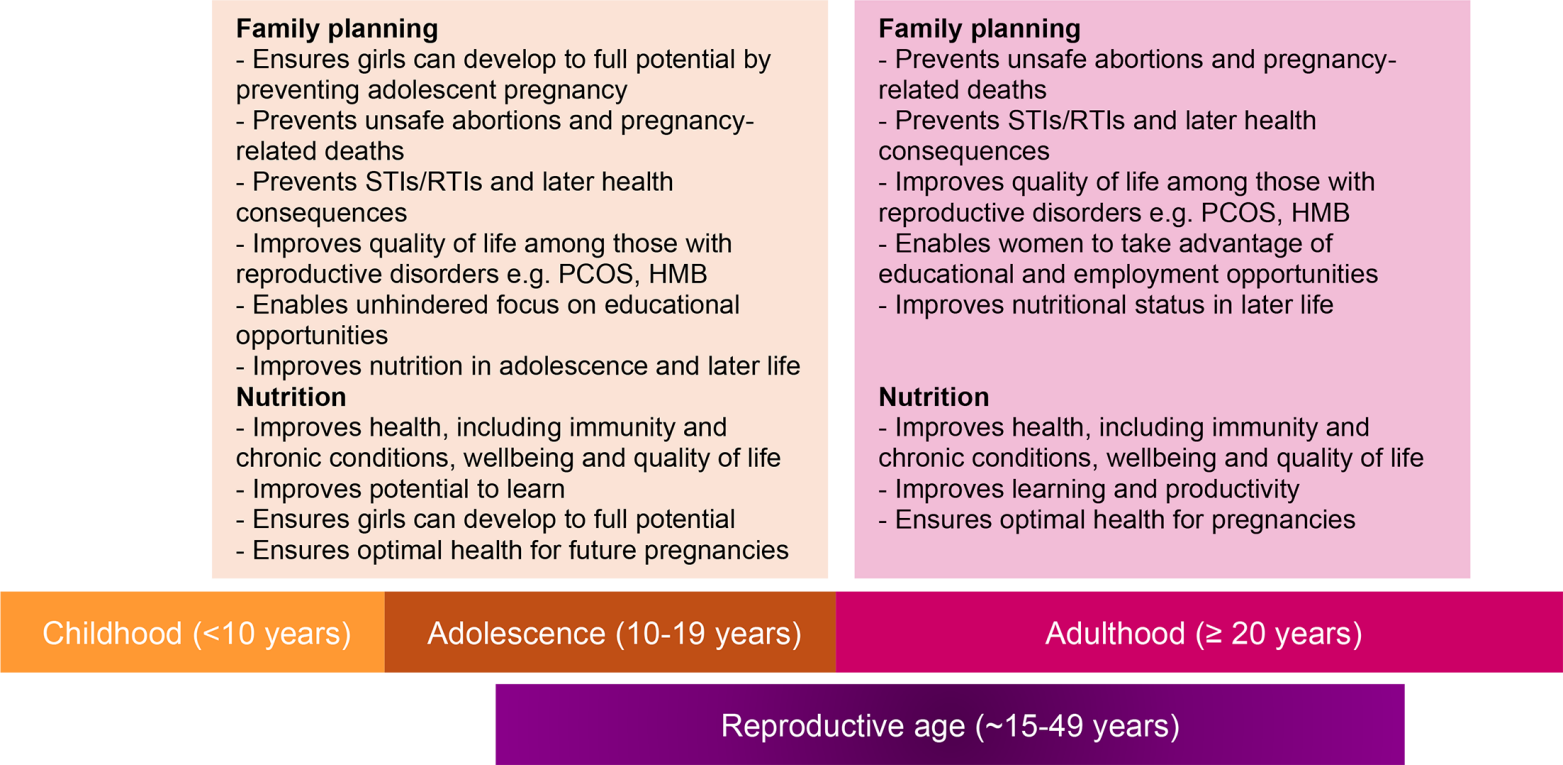
Co-occurrence of family planning and nutrition-related needs and benefits for women of reproductive age. HMB, heavy menstrual bleeding; PCOS, polycystic ovar y syndrome; RTI, reproductive tract infection; STI , sexually transmitted infection.

FP and nutrition-related needs co-occur in the same populations of WRA (figure 1). FP is needed once girls reach menarche and are sexually active, until menopause. Nutritional needs are substantial throughout adolescence and adulthood to facilitate physiological development and maintain optimal health, including before, during and after pregnancy. Optimising both FP and nutrition during adolescence and adulthood contributes to common pathways towards improving well-being throughout the life course, including protecting adolescent development, facilitating education and learning and preventing or managing reproductive conditions (figure 1).^9 10^

**Figure 2:**
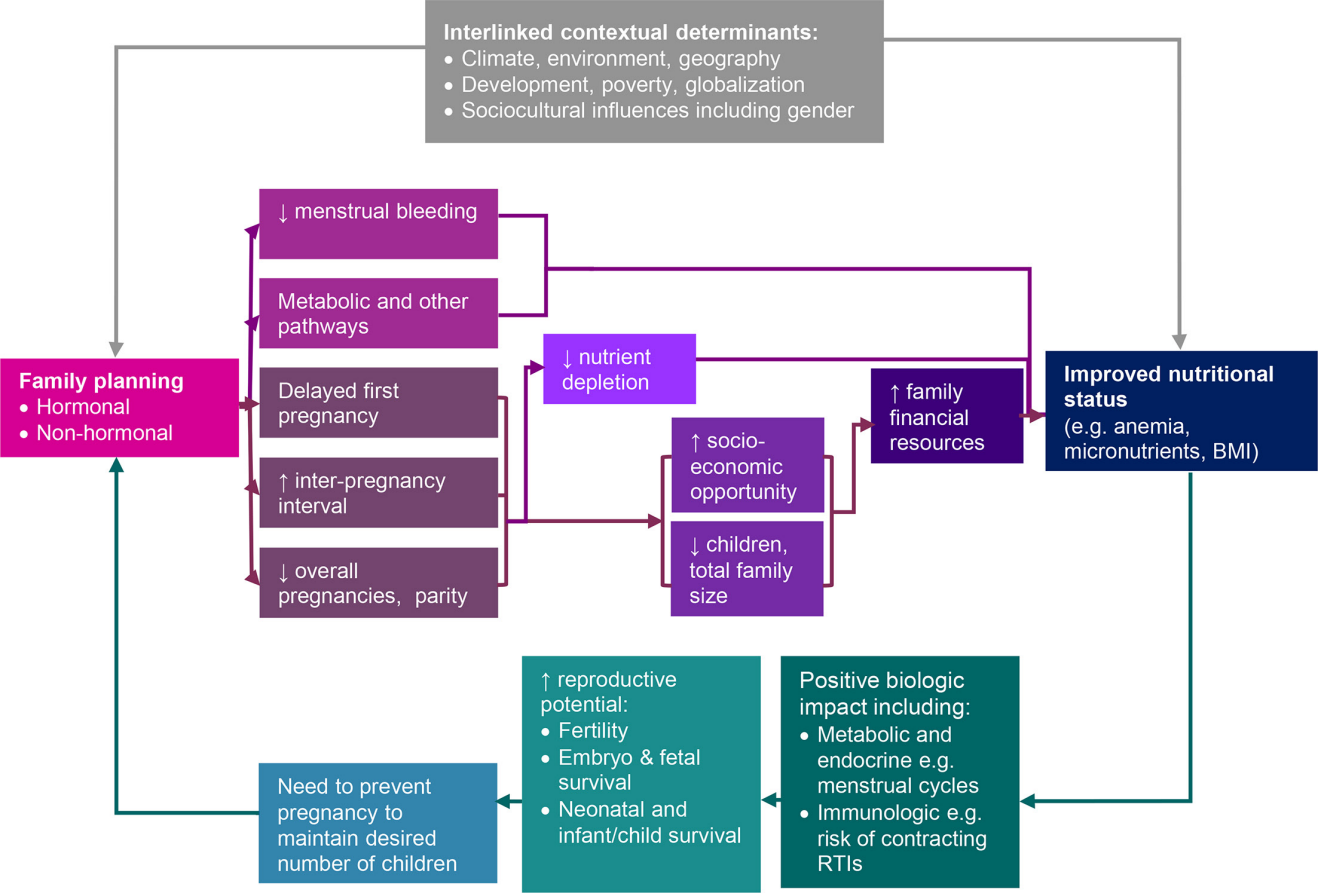
Conceptual framework of linkages between family planning and nutrition. BMI , Body Mass Index ; RTI , reproductive tract infection.

Furthermore, data indicate biological links between FP and nutritional status through multiple direct and indirect pathways (figure 2).^11^,^14^ Hormonal FP methods are understood to exert physiological effects on the body, leading to an impact on nutritional indices —for example, by reducing menstrual blood loss volume and thereby increasing haemoglobin.^1315^,^18^ Limited evidence also indicates alterations in micronutrient status (copper, zinc and vitamin D),^19 20^ as well as changes in weight ^21^ among women using hormonal contraceptives —although potential mechanisms have not been clearly outlined. Indirect links may be through control of the timing and number of pregnancies (figure 2). Pregnant adolescents face greater nutritional risks than pregnant adult women, including increased risk of anaemia in pregnancy and lower attained height, weight and fat mass accretion post-pregnancy and in adulthood.^22^,^27^ Closely spaced pregnancies are associated with maternal nutritional depletion, including a higher risk of anaemia during the next pregnancy ^28 29^ and congenital defects in offspring, reflecting low maternal folate and other micronutrient stores.^30^ Women with higher parity have been shown to have poorer micronutrient status, increased anaemia prevalence and lower height,^24 25 31^ and in high-income countries , high parity has been associated with later-life risk of increased BMI.^32^ Controlling the timing and number of pregnancies through FP enables women to avoid these nutritional risks. It may also enable WRA to take advantage of educational and employment opportunities, increase income ^33^ and benefit from increased family resources, with a resultant effect on access to adequate nutrition.^34 35^ On the other hand, nutritional status may also influence reproductive outcomes, including fertility and fetal survival, which could affect patterns of FP use in WRA (figure 2).^14^

Despite emerging evidence recognising these linkages, the available data h ave not been comprehensively summarised and considered in relation to FP-nutrition connections and how they could affect women’s health and well-being. We (the authors) recently undertook the Family Planning and Nutrition ( FPN) Project, which aimed to address this evidence gap by mapping the epidemiological and programmatic links between the two domains through a series of studies, focusing on LMICs. The project encompassed systematic and scoping reviews, stakeholder interviews, a survey of government officials, desk reviews and quantitative analyses examining data on ( 1) epidemiological links between FP and nutritional status; ( 2) integration of FP and nutrition services in LMICs and their impact and ( 3) perspectives on the value, opportunities and challenges of more closely aligning FP and nutrition services.^1314 36^,^40^ We draw on key findings from this project and related research to argue that FP-nutrition interlinkages have not been leveraged to their greatest potential due to a scarcity of robust evidence. We propose a multi-pronged research agenda to drive investments in improving women’s health through FP and nutrition and outline three priority areas for high-quality implementation studies: using FP as a tool to address anaemia, focusing on FP-nutrition service integration in the postpartum period and complementing social protection platforms addressing common factors influencing FP and nutrition.

## Using FP as a tool to address anaemia

Anaemia remains an important global health challenge, affecting a substantial proportion of WRA,^4^ leading to several adverse consequences for well-being and health, including reduced work productivity and adverse maternal and child outcomes.^41^ Much research has focused on micronutrient supplementation as a key strategy to address anaemia risk; however, micronutrient supplementation alone has shown limited success in substantially reducing anaemia.^42^,^45^ One reason for this may be that anaemia may have multiple determinants, including for WRA, heavy menstrual bleeding (HMB), which has a substantial estimated prevalence (over 40%) across regions and has been identified as an important chronic cause.^45 46^ Hormonal contraceptive methods, including the levonorgestrel-releasing intrauterine system (LNG-IUS) and oral contraceptives, form part of the clinical treatment recommendations for HMB ,^15^,^17^ with implications for reduced anaemia risk. The volume of cross-sectional data on associations between hormonal contraceptive use and increased haemoglobin is notable, and findings have also been replicated in some longitudinal observational and experimental studies.^13 15 16 18^ However, there is low certainty of evidence from experimental studies because of small sample sizes and the use of anaemia as a secondary measure.^13 18^ There is an opportunity to more robustly examine how FP can be leveraged as a tool to specifically address anaemia, with potentially important implications at the population level.

More robust evidence is required to inform potential investments in programmes that may leverage this important and direct FP-nutrition connection. We are aware of one ongoing study based in Kenya, examining the potential effectiveness of providing LNG-IUS to anaemic women compared with combined oral contraceptive pills, including ferrous fumarate.^47^ While this study will be key, it is important to be able to distinguish specifically the additional effect of hormonal contraceptives on anaemia status, particularly within the context of HMB. To address this, additional large-scale experimental studies are needed to examine the effect on anaemia of screening for and addressing HMB using hormonal contraceptive methods along with micronutrient supplementation versus no such screening or intervention (with or without supplementation).^19 48 49^ Such studies could capitalise on existing micronutrient supplementation infrastructure for WRA, where possible, to more clearly understand feasibility and related implementation indicators.^19 48 49^ Given sensitivities around the use of FP in certain settings and particularly among adolescent girls,^36^ studies could initially be based on populations where the use of FP (and hormonal contraceptives in particular) is more common and already generally accepted, including among populations of older WRA who have already met their childbearing goals or those presenting to health facilities with HMB as a primary complaint. These studies must also ensure that any potential causes of HMB are appropriately addressed, in line with established guidelines, including alternative treatment options where required.^50^ The initial goal of these studies should be to reliably ascertain the impact of FP-based approaches on anaemia in order to drive further investment; however, they must also measure effectiveness and cost-effectiveness, alongside potential side effects, to generate data to inform the expanded availability of FP commodities beneficial to nutritional status.

## Focusing on FP -nutrition service integration in the postpartum period

Alongside their biological interlinkages, FP and nutrition also hold importance across the same phases of life in WRA. There is thus a high value in aligning the delivery of FP and nutrition programmes to WRA to ensure that FP and nutritional needs are met at the same time, ensuring mutual reinforcement, maximising potential positive impact and increasing efficiency for both WRA beneficiaries and for health systems. Integrating services is also in line with the spirit of universal health coverage, including ensuring universal access to FP commodities and essential nutrition actions.^51 52^

In the FPN Project, the value of integrated FP-nutrition service delivery approaches was reflected in our qualitative study of global and regional FP, nutrition and related stakeholders, who highlighted the aforementioned advantages,^36^ and was further supported by country-specific mixed-methods investigations with WRA and community members, as well as country-specific policy and programme leaders in Tanzania and Burkina Faso.^38 39^ As part of these investigations and a scoping review of integrated FP and nutrition interventions,^37^ the pregnancy and postpartum period was commonly identified as an important and high-impact opportunity to achieve integration, given its importance to women’s and children’s health,^53^ as well as multiple existing health system contacts in this period.^36^ However, these studies, as well as a survey with health ministry officials from 64 countries across three WHO regions (Africa, South -East Asia and the Eastern Mediterranean), reiterate that FP and nutrition programmes are generally implemented separately.^38^,^40^ There is limited documentation of the integrated services across the life course, and even less data assessing benefits over and above standalone services on women’s FP and nutritional status in LMIC settings.^3754^,^56^ A key limitation to integrating services, as highlighted by stakeholders across studies in the FPN Project , was the lack of strong data on which integration models work, including demonstrations of their impact on women’s and children’s health, as well as on coverage and resource efficiency.^36 38 39^

The evidence above highlights that robust data on the benefits of FP-nutrition service integration are required to galvanise investments in population-level efforts by donors and governments. The FPN Project showed that the postpartum period is an opportunity for achieving integration. Alongside data indicating the benefits of lengthening inter-pregnancy intervals for women and children,^53^ growing evidence indicates that micronutrient supplementation during lactation improves maternal micronutrient status, with downstream benefits for infant nutrition.^57 58^ These findings support the examination of integrated postpartum FP and micronutrient supplementation as a more impactful approach than postpartum FP alone to improve maternal and child health and prepare women for subsequent pregnancies. Studies investigating combined postpartum FP and micronutrient supplementation approaches should capitalise on health system contacts throughout pregnancy and postpartum that are already mandated by policy to generate actionable indicators of impact and understanding of real-world implementation challenges.

Specific interventions to integrate FP and nutrition that are being tested as part of research efforts should be co-designed with local experts and communities to complement existing services and address additional human and other resource needs to ensure that health systems are not overburdened. Where possible, these interventions should build on ongoing efforts, such as the INSPiRE programme in West Africa, which opportunistically provides a comprehensive package of services, including FP to women from pregnancy to postpartum, and has been shown to increase service coverage.^59 60^ Research evaluating these interventions must also specifically quantify their impact on measures of maternal postpartum and child health and nutrition, and on subsequent pregnancy outcomes, alongside indicators of coverage, resources used, cost-effectiveness and transportability between rural/urban contexts. Successful postpartum FP-nutrition models could be used as a starting point to draw learnings for FP-nutrition interventions targeting additional defined phases of need, such as prior to the first pregnancy.

## Complementing social protection platforms, addressing common factors influencing FP and nutrition

FP use and nutrition not only affect each other in isolation but are driven by a cascade of multifactorial and interrelated determinants. Broader factors, including climatic and environmental conditions, affect economic and political stability, poverty and food insecurity (figure 2). These and sociocultural and gender norms influence household food allocation, women’s education and income-generating potential, age of marriage and, importantly, concepts of purity and stigma related to contraceptive use, especially among unmarried adolescent girls.^3661^,^64^ The impact of nutrition and FP interventions would most likely be greater when such contextual factors are addressed in tandem —yet there is limited evidence to drive investment in such efforts.

Social protection programmes address contextual factors determining access to FP and optimal nutrition ^65 66^ and provide excellent opportunities to deliver FP and nutrition interventions. These have the potential to be especially high-impact given that FP and nutrition -related needs are greatest among vulnerable populations.^38 39^ Previous research on the Oportunidades programme in Mexico and Bolsa Familia in Brazil has indicated positive effects of these conditional cash transfer programmes on contraceptive use, adolescent girls’ education and the reduction of adolescent pregnancy.^67 68^ Implementing and further building on such programmes with specific FP and nutrition services, such as contraceptive provision, referrals or food supplements, may provide a particularly strong opportunity to maximise benefits. This is supported by data from the survey with regional government officials, where respondents from over half of 64 countries indicated some provision of FP and nutrition services under social protection programmes for vulnerable groups.^40^ Robust evidence on the impact of co-delivering FP and nutrition services as part of existing social protection programmes could be measured using quasi-experimental or more robust stepped-wedge trials piloting such strategies. Such designs are particularly important given previous recognition of the limited reliable data to evaluate the impact of such programmes, which is understood to limit further investments.^67 68^

Efforts to co-deliver FP and nutrition services to women and girls as part of such programmes —whether counselling or commodities —will require effective approaches to address prevailing gender norms and stigma, particularly related to FP use.^36 69^ In line with this, stakeholders have highlighted the importance of ensuring that existing programmes are not compromised in coverage due to the addition of FP services.^36^ This reinforces the imperative of co-designing specific services and interventions with appropriate sensitisation, input and buy-in from the receiving communities. Ongoing projects will be key to driving learnings about how to overcome these barriers, such as the Building Rights for Improved Girl s’ Health in Tanzania programme, which aims to enable adolescent girls to exercise their rights related to optimal nutrition and sexual and reproductive health via engagement with family and key community members, including religious leaders and teachers, alongside improving service delivery.^56^ Sustained research and action addressing these upstream drivers will not only improve women’s access to FP and optimal nutrition but also more broadly address women’s rights, empowerment and well-being.

## Conclusions

Together with broader evidence, the FPN Project shows important epidemiological and programmatic linkages between FP and nutritional status that affect the health of WRA in LMICs.^1314 36^,^40^ Yet, important dimensions of these interlinkages remain underexploited, primarily because of limited evidence establishing the effectiveness of doing so. Here, we outline three potentially high-impact areas in which robust studies leveraging FP-nutrition links would provide the most valuable evidence to galvanise greater investment of resources in this area, including hormonal contraceptives for anaemia, postpartum FP-nutrition service integration and capitalising on social protection programmes. Continued effort to leverage FP-nutrition linkages in this way represents an important contribution to improving women’s health globally.

## Data Availability

No data were directly used in the preparation of this manuscript.
